# Control of Gene Expression With Quercetin-Responsive Modular Circuits

**DOI:** 10.3389/fbioe.2021.730967

**Published:** 2021-09-16

**Authors:** Fernanda Miyuki Kashiwagi, Brenno Wendler Miranda, Fabio de Oliveira Pedrosa, Emanuel Maltempi de Souza, Marcelo Müller-Santos

**Affiliations:** ^1^ Postgraduate Program in Science (Biochemistry), Department of Biochemistry and Molecular Biology, Nitrogen Fixation Laboratory, Federal University of Paraná (UFPR), Curitiba, Brazil; ^2^ Biological Sciences Undergraduate Course, Department of Biochemistry and Molecular Biology, Nitrogen Fixation Laboratory, Federal University of Paraná (UFPR), Curitiba, Brazil; ^3^ Nitrogen Fixation Laboratory, Department of Biochemistry and Molecular Biology, Federal University of Paraná (UFPR), Curitiba, Brazil

**Keywords:** genetic circuit, flavonoid, *E. coli*, quercetin, QdoR

## Abstract

Control of gene expression is crucial for several biotechnological applications, especially for implementing predictable and controllable genetic circuits. Such circuits are often implemented with a transcriptional regulator activated by a specific signal. These regulators should work independently of the host machinery, with low gratuitous induction or crosstalk with host components. Moreover, the signal should also be orthogonal, recognized only by the regulator with minimal interference with the host operation. In this context, transcriptional regulators activated by plant metabolites as flavonoids emerge as candidates to control gene expression in bacteria. However, engineering novel circuits requires the characterization of the genetic parts (e.g., genes, promoters, ribosome binding sites, and terminators) in the host of interest. Therefore, we decomposed the QdoR regulatory system of *B. subtilis*, responsive to the flavonoid quercetin, and reassembled its parts into genetic circuits programmed to have different levels of gene expression and noise dependent on the concentration of quercetin. We showed that only one of the promoters regulated by QdoR worked well in *E. coli*, enabling the construction of other circuits induced by quercetin. The QdoR expression was modulated with constitutive promoters of different transcriptional strengths, leading to low expression levels when QdoR was highly expressed and vice versa. *E. coli* strains expressing high and low levels of QdoR were mixed and induced with the same quercetin concentration, resulting in two stable populations expressing different levels of their gene reporters. Besides, we demonstrated that the level of QdoR repression generated different noise levels in gene expression dependent on the concentration of quercetin. The circuits presented here can be exploited in applications requiring adjustment of gene expression and noise using a highly available and natural inducer as quercetin.

## Introduction

Cells naturally sense and react to extracellular signals. This environmental computation is carried out by transcriptional regulators that increase or decrease gene expression upon the appearance of a new molecule in the extracellular medium (or a variation of its concentration). Synthetic biologists can arrange these natural transcriptional regulators with synthetic genetic elements in genetic circuits to explore new functionalities. Recently, several genetic circuits have been assembled and characterized, enabling cells to respond to non-cognate signals (Wang and Buck, 2012; Ma et al., 2016; Xia et al., 2019).

Some soil bacteria naturally recognize metabolites produced and emitted by plants in their root exudates. For instance, *Bacillus subtilis* has the TetR-type negative regulator QdoR that is induced by flavonoids such as quercetin and fisetin (Hirooka et al., 2007). Flavonoids are versatile plant secondary metabolites that defend plants from invaders and signal beneficial soil microorganisms. One of the most abundant flavonoids is quercetin, which is produced in root exudates of *Zea mays* (maize) (Kidd et al., 2001), *Arabidopsis thaliana* (Narasimhan et al., 2003), and *Alnus glutinosa* (Hughes et al., 1999). Quercetin inhibits the supercoiling activity of DNA gyrase B and induces DNA cleavage in bacteria, resulting in growth inhibition (Plaper et al., 2003). *B. subtilis* avoids the harmful effects of quercetin by expressing the quercetin 2,3-dioxygenase QdoI, which inactivates quercetin by converting it to 2-protocatechuoyl-phloroglucinol carboxylic acid and carbon monoxide (Hirooka et al., 2007). QdoR represses the QdoI expression, binding to specific operators upstream of *qdoI* (Hirooka et al., 2007). QdoR also interacts with an operator upstream of *qdoR,* repressing its own expression (Hirooka et al., 2007; Hirooka and Fujita, 2011). Quercetin inhibits the binding of QdoR to DNA; thus, the transcription of *qdoI* and *qdoR* is induced (Hirooka et al., 2007).

The QdoR regulatory system was applied to construct biosensors to detect the intracellular concentration of quercetin in *E. coli* (Siedler et al., 2014) and monitor the quercetin content in soil (Del Valle et al., 2020). However, there were only a few modifications in the genetic elements of the native system, which did not allow modulating the inducer-response curves. Genetic circuits to sense naringenin, which belongs to another class of flavonoids, have been more extensively engineered (De Paepe et al., 2018; Meyer et al., 2019; Ryu et al., 2020).

Expanding genetic circuits to various classes of flavonoids would give further alternatives for programming cells to respond to non-cognate signals. Aiming to build circuits activated by quercetin that can be transferred to bacteria that naturally do not have regulators that recognize these metabolites, we refactored the QdoR regulatory system of *B. subtilis,* combining it with synthetic promoters to modulate gene expression mediated by quercetin. The circuits built in this work can potentially be applied to control expression in complex environments such as soil and the rhizosphere surrounding the roots of plants.

## Materials and Methods

### *E. coli* Strains and Growth Conditions

*E. coli* TOP10 (Invitrogen, United States) was used for cloning purposes, while *E. coli* MG1655 (Blattner et al., 1997) was used as a chassis for testing the genetic circuits. The bacteria were grown in lysogeny broth (LB) (Sambrook and Russel, 2000) at 37°C, with shaking at 120 rpm (New Brunswick C25 Shaker), unless otherwise stated. The antibiotics were used in the following concentrations: ampicillin (250 μg ml^−1^) and chloramphenicol (25 μg ml^−1^).

### Plasmids Construction

The plasmids used in this study were constructed using the BioBrick assembly method (Shetty et al., 2008). The genetic parts were obtained from the Registry of Standard Biological Parts or designed to contain the prefix and suffix of BioBricks Standard Assembly (RFC 10). The transcriptional factor *qdoR*, with codons optimized for *E. coli* expression by JCat software (Grote et al., 2005), was synthesized on-demand (Genscript, United States) with the incorporation of the prefix and suffix of BioBricks Standard Assembly. The promoters of *qdoI* (P_*qdoI*_) and *qdoR* (P_*qdoR*_) were obtained by cloning of annealed oligos, with the incorporation of the prefix and suffix of BioBricks Standard Assembly (Table 1) into the EcoRI and PstI sites of pSB1C3. P_*qdoR*_ corresponds to the sequence between the nucleotides 4,107,952 and 4,107,995, and P_*qdoI*_ between the nucleotides 4,107,278 and 4,107,327 in the genome of *B. subtilis* 168 (Kunst et al., 1997). Seeking to make P_*qdoI*_ compatible with the Biobricks RFC10 assembly, an XbaI site in the wild-type sequence was mutated. Hence, P_*qdoI*_ was synthetized with the following substitutions: 41 G > C and 42A > T. Both substitutions lay between the -35 and -10 sites and outside the QdoR operators (Supplementary Table 1). From the Registry of Standard Biological Parts, we used synthetic constitutive promoters (BBa_J23114, BBa_J23115, BBa_J23116, BBa_J23105, and BBa_J23110), an RBS (BBa_B0034), a transcription terminator (BBa_B0015), and composite parts (BBa_I13504 and BBa_K1357010) formed by an RBS, *gfp* or *rfp* genes, and a transcription terminator. All the plasmids (Table 1) were constructed using pSB1C3 or pSB1A2 as backbone vectors (Registry of Standard Biological Parts), containing a pUC19-derived high copy replication origin and a chloramphenicol or ampicillin resistance marker. The DNA sequences of the genetic parts used in this work are provided in the Supplementary Material.

**TABLE 1:** Plasmids constructed and used in this study.

*E. coli*	Relevant characteristics	References
TOP10	Cloning strain	Invitrogen
MG1655	*E. coli* K-12-derivative strain	Blattner et al. (1997); Soupene et al. (2003)
**Plasmid**		
pSB1C3	High copy number plasmid for Biobricks assembly (standard RFC [10]) carrying chloramphenicol resistance	iGEM repository
pSB1A2	High copy number plasmid for Biobricks assembly (standard RFC [10]) carrying ampicillin resistance	iGEM repository
pFMK1	Cm^R^, P_*qdoR*_-RBS-*qdoR*-T- P_*qdoI*_-RBS-*gfp*-T	This work
pFMK2	Cm^R^, P_*qdoR*_-RBS-*qdoR*-T- P_*qdoR*_-RBS-*gfp*-T	This work
pFMK3	Cm^R^, P_*J23114*_-RBS-*qdoR*-T- P_*qdoI*_-RBS-*gfp*-T	This work
pFMK4	Cm^R^, P_*J23115*_-RBS-*qdoR*-T- P_*qdoI*_-RBS-*gfp*-T	This work
pFMK5	Cm^R^, P_*J23116*_-RBS-*qdoR*-T- P_*qdoI*_-RBS-*gfp*-T	This work
pFMK6	Cm^R^, P_*J23105*_-RBS-*qdoR*-T- P_*qdoI*_-RBS-*gfp*-T	This work
pFMK7	Cm^R^, P_*J23110*_-RBS-*qdoR*-T- P_*qdoI*_-RBS-*gfp*-T	This work
pFMK8	Cm^R^, P_*J23114*_-RBS-*qdoR*-T- P_*qdoI*_-RBS-*rfp*-T	This work
pFMK9	Cm^R^, P_*J23110*_-RBS-*qdoR*-T- P_*qdoI*_-RBS-*rfp*-T	This work
pFMK10	Amp^R^, P_*J23114*_-RBS-*gfp*-T	This work
pFMK11	Cm^R^, P_*qdoI*_-RBS-*gfp*-T	This work
pFMK12	Cm^R^, P_*J23110*_-RBS-*qdoR*-T- P_*qdoR*_-RBS-*gfp*-T	This work

P_subscript_, the subscript refers to the promoter sequence; RBS, B0034 BioBrick code; T, B0015 BioBrick code; *gfp*, gene expressing the GFPmut3b variant of GFP; *rfp*, gene expressing the mRFP variant of DsRed.

### Cell Fluorescence Measurements

*E. coli* MG1655 cells transformed with a plasmid from Table 1 were inoculated and grown overnight at 37°C and 120 rpm. These cultures were diluted (1:100) in 200 µL of fresh LB medium and incubated in 96-well plates (Greiner Bio-One, 96 Flat Clear Bottom Black Polystyrene) to an optical density at 600 nm (OD_600_) of approximately 0.7. Then, different concentrations of quercetin dissolved in dimethylsulfoxide (DMSO) were added. Fluorescence was followed during incubation at 37°C using two different methods: 1) culture directly in a fluorescence plate reader and 2) culture in 96-well plates with incubation in a shaker and cell fluorescence analyzed by flow cytometry.

The fluorescence plate reader was a Tecan Infinite 200 (Tecan, Switzerland). Cultures were performed directly in the reader with 5 mm orbital shaking for 6 h after induction with hourly measurements of fluorescence and OD_**600**_. GFP fluorescence was measured with an excitation wavelength (λ_ex_) of 485 nm and an emission wavelength (λ_em_) of 535 nm with the gain set at 115 unless otherwise stated.

The flow cytometry measurements were done by first incubating the induced cultures for 4 h, centrifugation of 1 ml of culture (12,000 x g, 1 min, RT), and cell resuspension with TBAC buffer (PBS buffer containing 1 mm EDTA and 0.01% (v/v) Tween 20). The GFP fluorescence was measured in a BD Accuri™ C5 flow cytometer (San Jose, CA, United States) with a 488 nm longpass and a 533/30 nm bandpass filter set. Data were analyzed using FlowJo™ software to obtain FL median (the fluorescence median intensity) and coefficient of variation (CV) values. The CV was calculated as follows:

CV (%)  =  (standard deviation of the sample)/mean × 100.

### Fluorescent Measurement With Mixed *E. coli* Cultures

*E. coli* MG1655 cells transformed with plasmids carrying either *gfp* or *rfp* were separately inoculated and grown at 37°C and 120 rpm. The cultures were diluted 100-fold in LB medium (10 ml) and incubated in 60 ml flasks until OD_600_ reached ∼0.8. Each culture was diluted to OD_600_ of 0.6, and both cultures (one carrying *gfp* and another *rfp*) were mixed in equal proportion. 200 µL of the mixture was transferred to 96-well plates (Greiner 96 Flat Bottom Black Polystyrene) and increasing concentrations of quercetin in DMSO were added. Only DMSO was added to the uninduced control. The cultivation and fluorescence measurement were carried out as mentioned above. GFP fluorescence was measured with an excitation wavelength of 485 nm and an emission wavelength of 535 nm. RFP fluorescence was measured with an excitation wavelength at 540 nm and emission wavelength of 650 nm.

### Hill Fitting and Statistical Analysis

The fluorescence data for each concentration of quercetin were fitted with the Hill function, as follows:

$$\left(\frac{\text{FL}}{{\text{OD}}_{600}}\right)={\text{y}}_{0}+\frac{\text{β}{\left[\text{Q}\right]}^{\text{n}}}{\left({\left[\text{Q}\right]}^{\text{n}}+{\text{K}}_{0.5}^{\text{ n}}\right)},$$

where FL/OD_600_ is the specific fluorescence, y_0_ is the basal specific fluorescence, β is the relative maximum specific fluorescence, [Q] is the quercetin concentration in µM, n is the Hill coefficient, and K_0.5_ is the Hill constant (half-maximal quercetin concentration, µM). The fitting was carried out using the Solver function in Microsoft Excel®. Statistical analyses were carried out using the independent two-sample *t*-test with the R package (R Core Team, 2020).

## Results

### Comparing the Regulation of Two Promoters Repressed by QdoR

**FIGURE 1:**
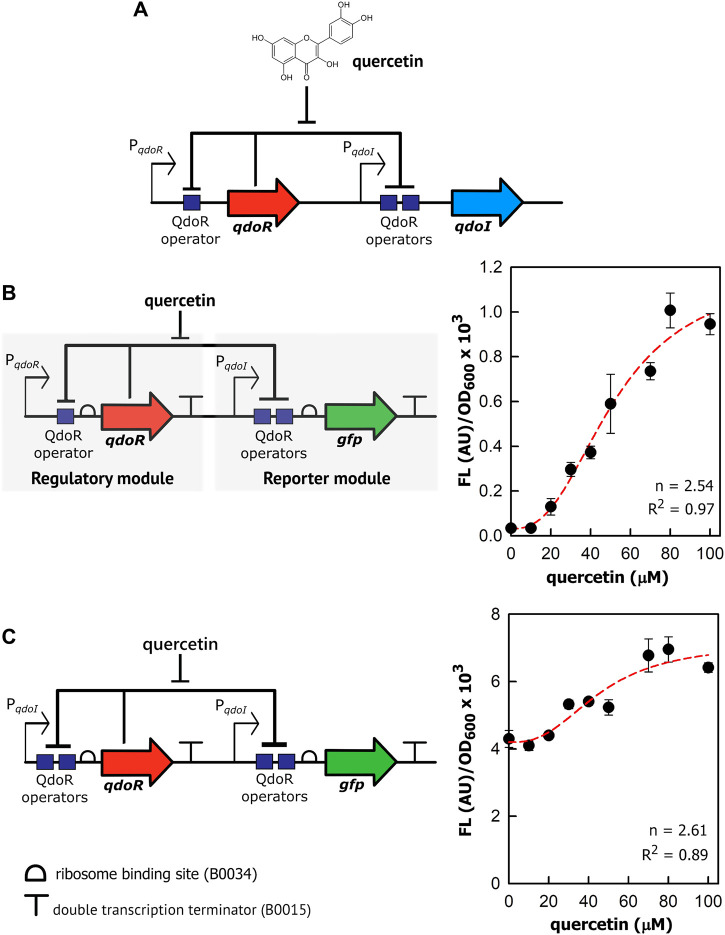
Quercetin induces gene expression in the reassembled genetic circuits repressed by QdoR. **(A)** The natural QdoR/P_*qdoI*_ system of *Bacillus subtilis*. The *qdoR* expression is self-regulated by QdoR. The P_*qdoR*_ promoter has one QdoR operator downstream of the RNA polymerase -10 recognition site. QdoR binds to two operators in the P_*qdoI*_ promoter, repressing *qdoI* expression. Quercetin binds to QdoR, reducing its affinity for DNA and derepressing *qdoR* and *qdoI* expression. **(B)** Genetic circuit constructed with minimal promoters P_*qdoR*_ and P_*qdoI*_ controlling *qdoR* and *gfp* expressions, respectively. The regulatory module controls the expression of QdoR, while the reporter module controls the expression of the reporter GFP. **(C)** Alternative circuit with *qdoR* under control of P_*qdoI*_. The graphs at the right of the circuit schemes show the specific GFP fluorescence in arbitrary units (FL (AU)) normalized by OD_600_ of the *E. coli* culture as a function of quercetin concentration added to the medium. The FL and OD_600_ shown in the graphs were measured after 6 h of growth. About that time after induction, FL/OD_600_ values were already stable (Supplementary Figure S5). The red dashed lines represent the Hill function fitting to the experimental data. The n and *R* ^2^ values are the Hill coefficient and coefficient of determination, respectively, for each plot. Note that the genes and regulatory elements in the circuit schemes are not to scale. The sequences of the genetic part assembled in the circuits are provided in Supplementary Table S1. The experiments were conducted with biological triplicates. After induction, the fluorescence of each replicate was measured once at specified times. The error bars represent the standard deviation.

To construct genetic circuits responsive to quercetin, we first dissected the QdoR regulatory system of *B. subtilis,* isolating *qdoR*, *qdoI*, QdoR operators, and their promoters (Figure 1A). We reassembled them with synthetic parts (constitutive promoters, a ribosome binding site, and a transcription terminator) and cloned them in plasmids to transform *E. coli*. The sequence of each genetic part used is provided in Supplementary Table 1. Firstly, we cloned *qdoR* under the control of its promoter (P_*qdoR*_) and the reporter *gfp* under the control of the *qdoI* promoter (P_*qdoI*_). Both genes have a synthetic RBS (B0034) at their 5′ flank and a double terminator (B0015) at 3’ flank. Quercetin induced GFP expression in *E. coli* (Figure 1B), demonstrating that the P_*qdoR*_-*qdoR*-P_*qdoI*_-*gfp* circuit was responsive between 20 and 80 µM quercetin. The quercetin induction increased the specific fluorescence intensity (Fluorescence/OD_600_) 33-fold, representing a 4.7-fold increase in the lowest to highest reporter expression compared to a previous quercetin biosensor in *E. coli* (Siedler et al., 2014). The Hill coefficient for P_*qdoR*_-*qdoR*-P_*qdoI*_-*gfp* circuit was 2.54 (*R* ^2^ = 0.97), displaying a cooperative and ultrasensitive response (Bradley et al., 2016).

As reported by Hirooka (2007), the *qdoI* promoter has two QdoR operator sites, whereas only one operator was identified upstream *qdoR*. In turn, to check whether a more repressed state with two operators could amplify the output upon quercetin induction, we rearranged the circuit components putting *qdoR* under the control of the *qdoI* promoter (Figure 1C). For the sake of clarity, hereafter, we shall refer to input as the quercetin concentration added to the system and output as the fluorescence level generated by GFP or RFP expression. Although quercetin induced GFP expression, a high basal fluorescence was measured in uninduced *E. coli*, indicating leakage of the P_*qdoI*_ controlling the reporter. The basal fluorescence was 130 times higher than that in the negative regulated P_*qdoR*_-*qdoR*-P_*qdoI*_-*gfp* circuit. The specific fluorescence increased only 1.8-fold from 10 to 80 µM quercetin. The Hill coefficient to this plot was 2.61; however, the data from this circuit did not fit so well the sigmoidal function (*R* ^2^ = 0.89).

The P*qdoI* controlling *qdoR* should be more repressed by QdoR than P*qdoR*, which decreased the QdoR concentration in *E. coli* and made the reporter module a little repressed. In contrast, P_*qdoR*_ was unable to control the reporter module, even when we put *qdoR* under the control of a strong constitutive promoter, J23110, as the GFP expression controlled by P_*qdoR*_ was completely derepressed and non-responsive to quercetin (Supplementary Figure S2). Even adding quercetin above 100 µM to *E. coli* with the P_*qdoR*_-*qdoR*-P_*qdoI*_-*gfp* circuit, we did not reach the maximum specific fluorescence produced by the P_*qdoI*_-*qdoR*-P_*qdoI*_-*gfp* circuit. Likely, the circuit P_*qdoR*_-*qdoR*-P_*qdoI*_-*gfp* forms negative feedback induced by quercetin, resisting to express more GFP as more quercetin is added. On the other hand, the P_*qdoI*_-*qdoR*-P_*qdoI*_-*gfp* circuit leaves the reporter module almost completely unrepressed due to low QdoR expression. In agreement, the basal specific fluorescence of P_*qdoI*_-*qdoR*-P_*qdoI*_-*gfp* is high ∼4×10^3^ AU/OD_600_. Of note, quercetin uptake has already been studied in *E. coli* W3110, showing that in a solution with 69 µM quercetin, the intracellular quercetin concentration was stable at ∼2.5 µM from 2 to 6 h of incubation (Said et al., 2016). We did not notice the growth effect in *E. coli* MG1655 with quercetin from 10 to 100 (data not shown).

In summary, two operator boxes, as given by the *qdoI* promoter, are necessary to increase repression and control the reporter module. On the other hand, the weak repression in the *qdoR* promoter is necessary to maintain the QdoR levels to control both regulatory and reporter modules. Moreover, manipulating the QdoR expression would make it possible to tune circuits to give different outputs.

### The Constitutive Expression of QdoR With Synthetic Promoters Creates Circuits That Generate Distinct Outputs for the Same Input

**FIGURE 2:**
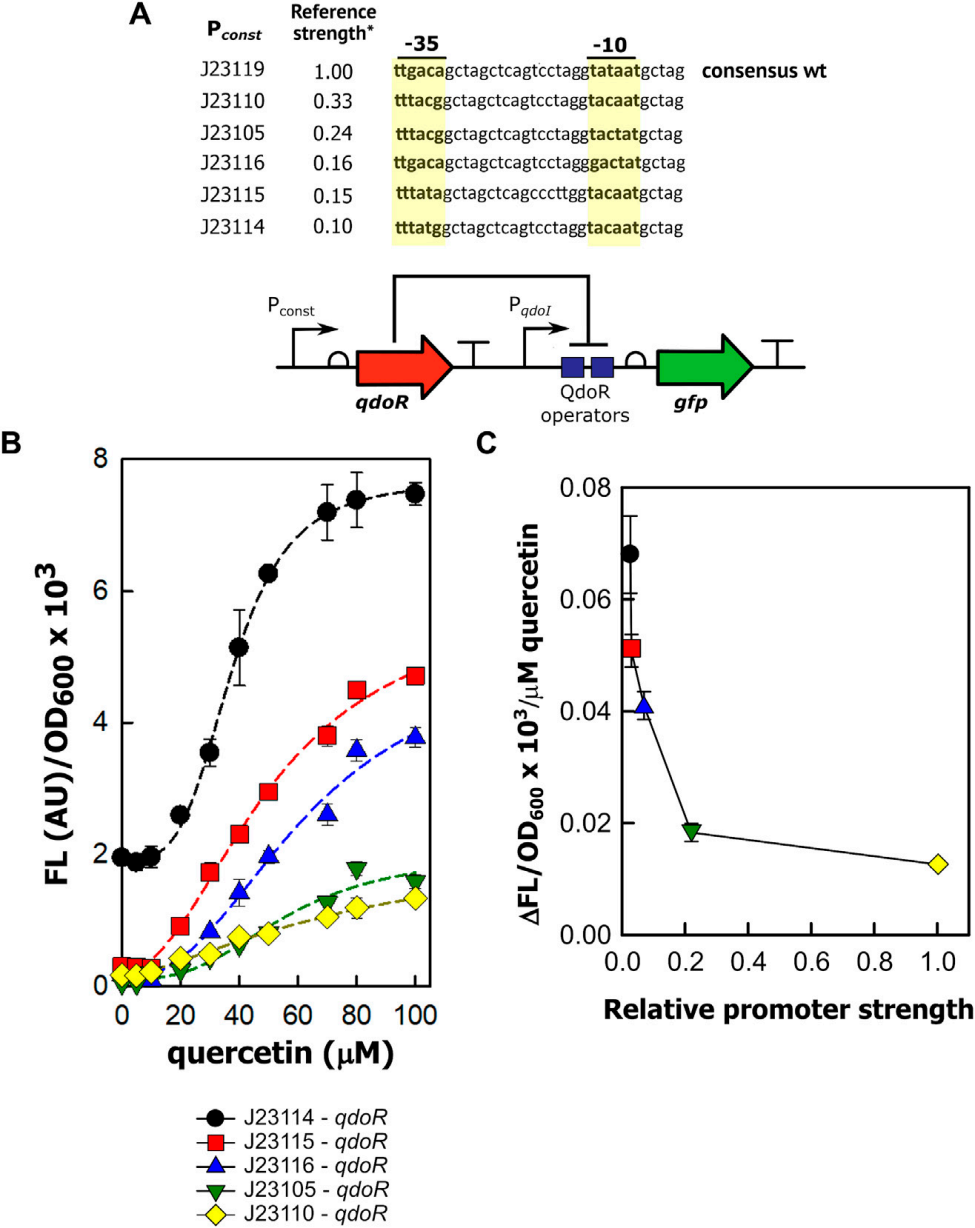
The expression of QdoR can be adjusted to give staggered outputs. **(A)** In five different constructions, constitutive promoters (P_*const*_) with different transcriptional strengths were inserted upstream of *qdoR*. The promoter sequences are shown, with the −35 and −10 recognition sites in bold and highlighted in yellow. The top sequence is referred to as the promoter id J23119. *The reference promoter strengths were reported previously in the iGEM repository dataset and indicate the strength expected to each promoter selected to construct the circuits. **(B)** The outputs of the circuits (FL/OD_600_) were plotted as a function of quercetin concentration added to the medium. The FL/OD_600_ showed in the graphs were measured after 6 h of growth. About that time after induction, FL/OD_600_ values were already stable (Supplementary Figure S5). The symbols below the graph denote the circuits evaluated in the experiment. The dashed lines represent the Hill function fitting to the experimental data. The n and *R* ^2^ coefficients for each plot are presented in Table 2. **(C)** We applied linear fit to the curves in B between 10 and 80 µM quercetin. The values of ΔFL/OD_600_×10^3^/µM quercetin were plotted against the experimental strengths of the promoters, relative to that of J23110 (which therefore has a relative strength of 1). The promoter strengths were obtained experimentally measuring specific fluorescence (FL/OD_600_) during exponential growth of *E. coli*; the data are provided in Supplementary Figure S1. The GFP expression was measured as output. The symbols for each circuit were plotted following the same scheme as that of graph B (see the symbols below graph B). The experiments were conducted with biological triplicates. After induction, the fluorescence of each replicate was measured once at specified times. The error bars represent the standard deviation.

We then investigated whether we could get staggered outputs in circuits with the level of QdoR adjusted using constitutive promoters of medium and low transcription strength. By staggered outputs, we mean that the maximum GFP expression of these circuits will reach intermediate values, smaller than those for the unrepressed circuit (P_*qdoI*_-*gfp*). The circuits were designed with non-feedback regulation since the QdoR expression does not depend on the quercetin concentration. Similar arranges expressing the TetR repressor constitutively were applied to evaluate the output levels and noise generated in *E. coli* (Dublanche et al., 2006) and *Saccharomyces cerevisiae* (Nevozhay et al., 2009). Although negative autoregulation has been used to adjust inducer-output response curves and reduce noise in expression (Becskel and Serrano, 2000; Dublanche et al., 2006; Nevozhay et al., 2009), if we apply a circuit where QdoR represses its own expression and the reporter simultaneously, we will obtain a closed loop giving a single input-output response upon induction. On the other hand, by analogy with Ohm’s law (V = I × R), if we design circuits with different resistances (QdoR expression, analogous to the resistance R), they should respond with different outputs (GFP expression, analogous to the current I) to the same input (quercetin concentration added, analogous to the applied voltage). To verify this hypothesis, we put *qdoR* under the control of five promoters with reference relative expression strengths ranging from 0.10 to 0.33 (Figure 2A, Supplementary Figure S1). The relative strengths were reported previously by J. Christopher Anderson and are available at the iGEM repository dataset (http://parts.igem.org/Promoters/Catalog/Anderson). However, as we cannot guarantee that our experimental conditions were the same as those used by J. Christopher Anderson, we determined the strengths of the synthetic promoters and the P_*qdoI*_ and P_*qdoR*_ promoters (Supplementary Figure S1). All the circuits detected a minimum concentration of 20 µM quercetin, and the highest fluorescence was reached with 80–100 µM (Figure 2B). The circuit with the lowest resistance (J23114-*qdoR*) had a 3.5-fold increase in the dynamic range with 80 µM of inducer and the highest cooperativity (*n* = 3.79, Table 2). The maximum output decreased with increasing resistance in J23115-*qdoR* and J23116-*qdoR* circuits, but they had lower reporter leakage in the uninduced state, comparing y_0_ values in Table 2. Although the output gain between the uninduced and induced states was 15-fold, the cooperativity was reduced (J23115-*qdoR*, *n* = ∼2.1; J23116-*qdoR*, *n* = ∼2.4). However, in J23105-*qdoR* and J23110-*qdoR*, where the promoter controlling the circuit had in our experimental conditions 6 and 37% of the strength of a sigma 70 consensus promoter in *E. coli* (J23119), respectively (Supplementary Figure S1), although the circuit was still inducible, it tended to be locked into a non-activable state; 100 µM of inducer gave only 18% of the maximum output measured for the less resistive circuit with the lowest *qdoR* expression, J23114-*qdoR* (Figures 2B, C). The medium resistance circuits (J23115-and J23116-*qdoR*) gave maximum outputs that were around 3-fold higher than those obtained with the higher resistance circuits (J23110-and J23105-*qdoR*). The cooperativity was also severely reduced (*n* = ∼1.5). The J23119-*qdoR* circuit showed a low derepression even above 50 µM quercetin (the maximum output was 0.8 × 10^3^/OD_600_, Supplementary Figure S3).

**TABLE 2:** Data fitting for the parameters of the circuits induced by quercetin using Hill function.

Circuit	y_0_ (×10^3^)	β (×10^3^)	K_0.5_ (µM)	n	*R* ^2^
P_*qdoR*_-*qdoR*-P_*qdoI*_-*gfp*	0.03	1.14	51.98	2.54	0.97
P_*qdoI*_-*qdoR*-P_*qdoI*_-*gfp*	4.20	6.77	45.61	2.61	0.89
J23114-*qdoR*-P_*qdoI*_-*gfp*	1.94	7.48	37.60	3.79	0.99
J23115-*qdoR*-P_*qdoI*_-*gfp*	0.23	4.74	51.30	2.09	0.99
J23116-*qdoR*-P_*qdoI*_-*gfp*	0.12	3.82	63.48	2.37	0.98
J23105-*qdoR*-P_*qdoI*_-*gfp*	0.11	1.74	56.16	2.90	0.96
J23110-*qdoR*-P_*qdoI*_-*gfp*	0.15	1.33	82.72	1.45	0.99

y_0_, basal output; β, maximum output; K_0.5_, quercetin concentration to reach half of the maximum output; n, Hill coefficient; *R* ^2^, coefficient of determination.

In summary, non-feedback circuits based on QdoR can exploit constitutive promoters with transcription forces less than 25% of the strongest consensus promoter, J23119. Above that, the amount of QdoR per cell would become excessive, blocking all QdoR operator sites in P_*qdoI*_, even with a high inducer concentration.

### Circuits With High Resistance Are Prone to Be Noisy

**FIGURE 3:**
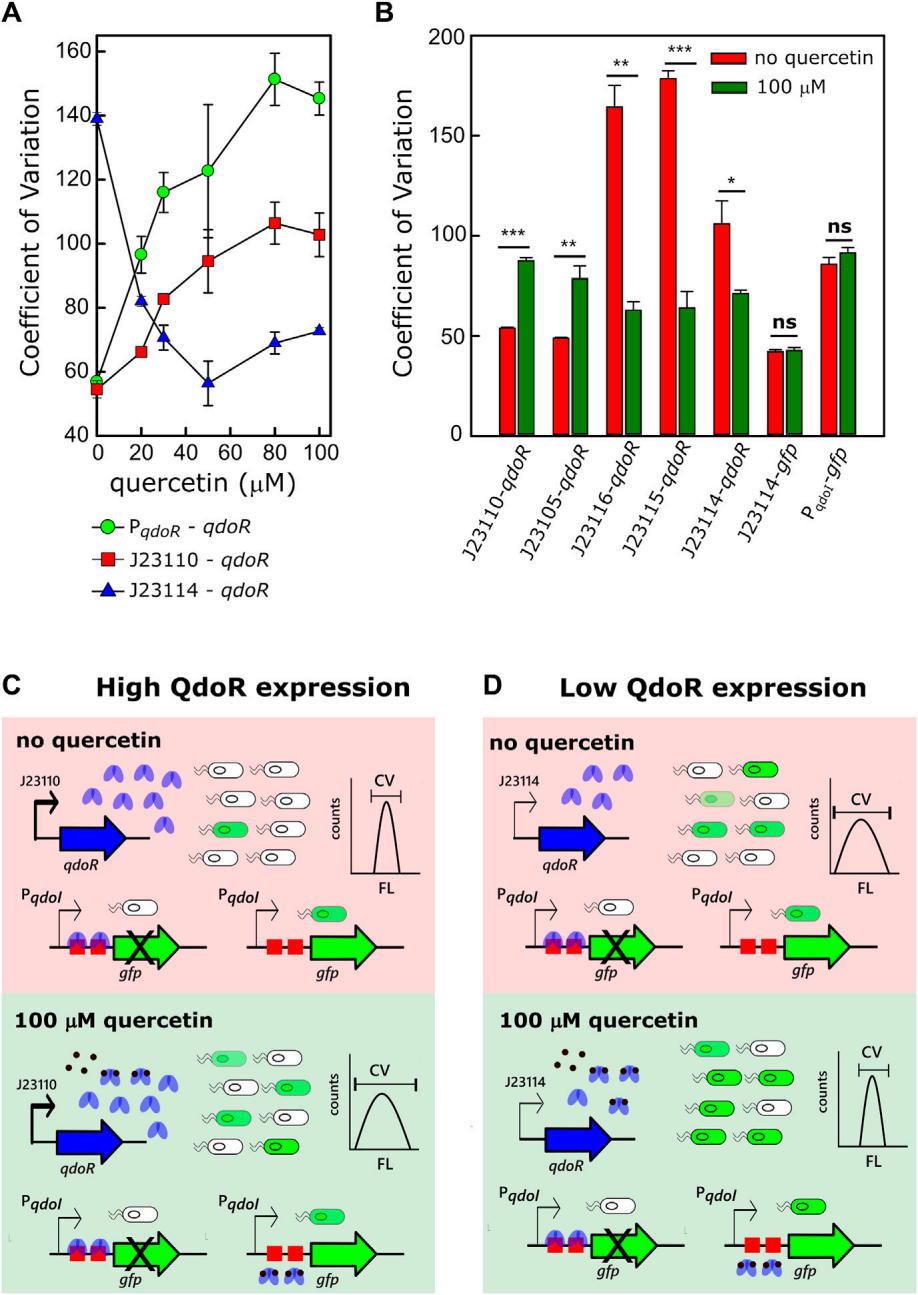
The QdoR expression level affects the amount of noise upon quercetin induction. **(A)** The coefficient of variation (CV) was calculated from the FL values measured by flow cytometry (λ_ex_ = 488 nm, λ_em_ = 530 nm). Three circuits controlling *gfp* expression were induced with increasing quercetin concentrations: P_*qdoR*_-*qdoR* (*qdoR* expression controlled by the minimal *qdoR* promoter) and J23110-*qdoR* and J23114-*qdoR* (*qdoR* is expressed constitutively by the J23110 and J23114 promoters, respectively). The experiment was conducted with biological triplicates. **(B)** The CV of GFP expression in uninduced and induced *E. coli* cultures was correlated with the circuits depicted in Figure 2A; all these circuits had *gfp* as a reporter. The J23114-*gfp* construct has the J23114 constitutive promoter controlling the expression of *gfp*. The P_*qdoI*_-*gfp* construct has the non-repressed *qdoI* promoter controlling *gfp* expression. Note that both circuits without QdoR repression do not have considerable variations on CV values upon the addition of quercetin. **(C, D)** Illustrative representation of the effect of QdoR expression on the CV values of GFP expression. Two states are considered in either high QdoR expression (J23110-*qdoR*) or low QdoR expression (J23114-*qdoR*): in light red, the uninduced repressed circuit, in light green, the circuit induced with 100 µM quercetin. J23110-*qdoR* and J23114-*qdoR* represent the regulatory modules. Below them, the possibilities of outputs to the reporter modules are depicted. White bacteria are completely repressed by QdoR with no GFP expression, while green bacteria have some GFP expression level that increases when quercetin is added. Note that the number of QdoR is high when J23110 controls the circuit, leading to derepression resistance when quercetin is added and an increase in noise (high CV amplitude in counts against FL plot). The cytometric profiles (counts vs. fluorescence intensity) to each circuit at the uninduced and induced states are provided in Supplementary Figure S4 and Supplementary Table S3. Statistical significance of the comparison for each circuit in uninduced and induced states is shown as *p*-values* ≤ 0.05, ** *p*-value ≤ 0.01, **p-value ≤ 0.001 (independent two-sample *t*-test). The statistical comparisons between all circuits are provided in Supplementary Table S2. The experiments were conducted with biological triplicates. After induction, the fluorescence of each replicate was measured once at specified times. The experiments were conducted with biological triplicates. After 4 h of induction, the fluorescence of each replicate was measured once. The error bars represent the standard deviation.

Variation in transcription is a significant factor in generating gene expression noise (Raj and van Oudenaarden, 2008). Considering that the constitutive promoters controlling *qdoR* used in this work have a wide range of transcription strength, it is likely that some of the non-feedback circuits expressing QdoR would be noisy. We evaluated gene expression noise with the coefficient of variation (CV), given by the ratio of the standard deviation to the mean. The CV values were obtained by flow cytometry analysis of the fluorescence intensity of *E. coli* cells carrying each expression circuit. For the circuit with negative feedback by QdoR (P_*qdoR*_-*qdoR*), the CV increased 2.5-fold between the uninduced and the fully induced state with 80 µM of quercetin (Figure 3A). The same increase in CV was also obtained for the circuit with higher resistance (J23110-*qdoR*) when quercetin was added to the system. In the same way, the CV in the induced state was twice the CV in the uninduced state. Interestingly, for the lower resistance circuit (J23114-*qdoR*), the CV decreased upon induction, reducing 2.3-fold with 50 µM of quercetin.

We extended the analysis to the other circuits and found that for the circuits with higher resistance to derepress GFP expression (J23110-*qdoR* and J23105-*qdoR*), the CV increased from the uninduced to the fully induced state. For the medium resistance circuits (J23115-*qdoR* and J23116-*qdoR*), induction reduced the CV by about 3-fold (Figure 3B, Supplementary Figure S4).

Under high repression (Figure 3C), when reporter expression is induced, many QdoR molecules can still bind many operators and block transcription of *gfp*. Therefore, a small number of cells within the clonal population can reach a derepression level sufficient to express some GFP. An initial highly repressed state makes the population more homogeneous but making it noisy when the input comes. Under low repression (Figure 3D), the scenario is the reverse; there are too few QdoR molecules to repress GFP expression entirely in the uninduced state, which leads to noise. Induction increases the homogeneity of the population since there are sufficient quercetin molecules to bind most of the QdoR molecules. This behavior is what Ozbudak et al. referred to as translational burst, which occurs when a cell population has a low transcriptional rate but a high translational rate (Ozbudak et al., 2002).

### Mixed Bacterial Cultures Carrying Circuits That Express Different Levels of QdoR Behave Independently for the Same Input

**FIGURE 4:**
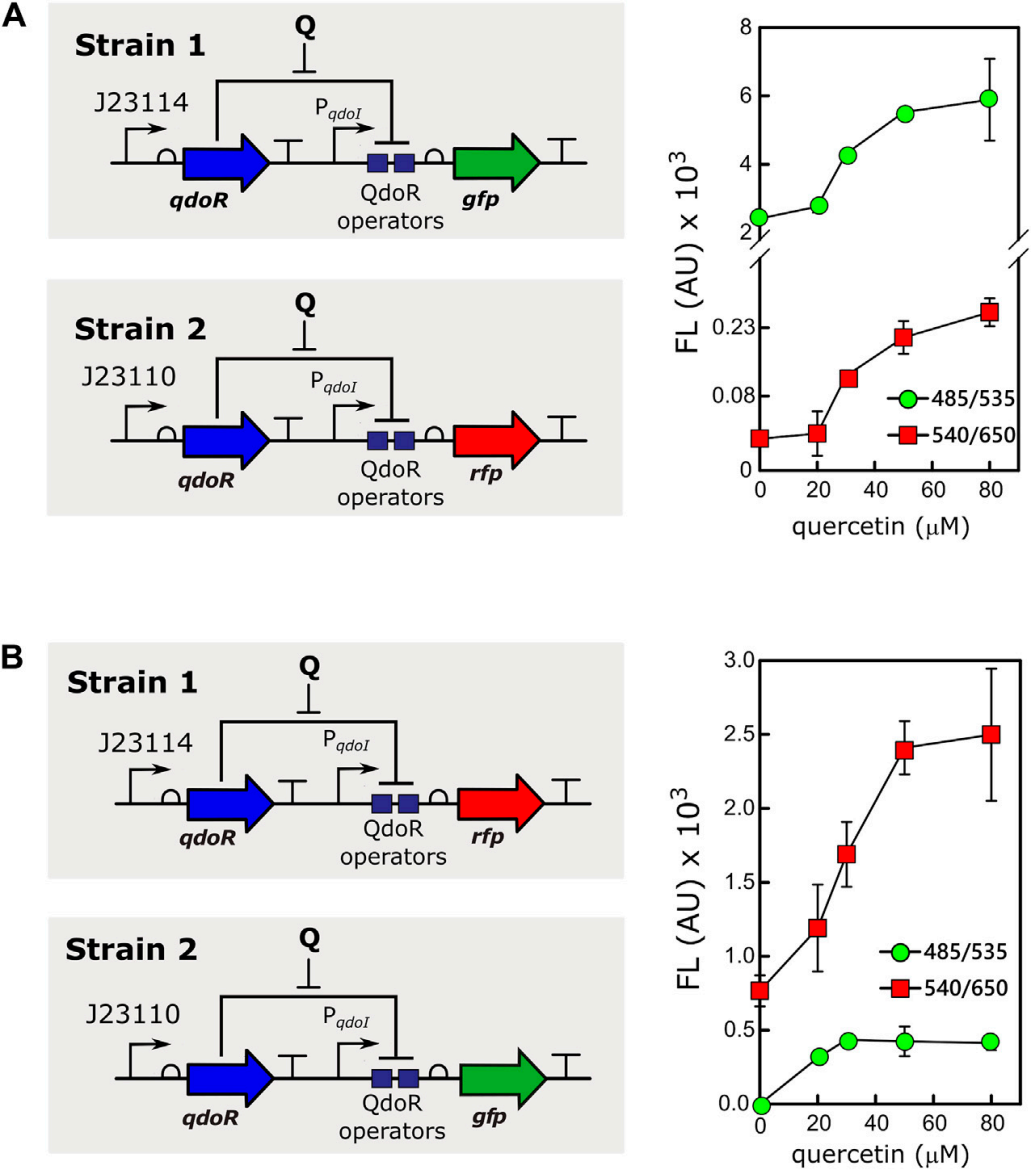
The same inducer concentration gives different outputs in a mixed culture of *E. coli* strains. **(A)** Strain 1, *E. coli* MG1655 carrying the circuit J23114-*qdoR*-P_*qdoI*_-*gfp* was mixed with strain 2, *E. coli* MG1655 with J23110-*qdoR*-P_*qdoI*_-*rfp*, keeping an equal proportion of both strains. The mixed culture was induced with different concentrations of quercetin, and fluorescence (FL) was measured using the following combinations of emission (λ_em_) and excitation (λ_ex_) wavelengths: 485 nm (λ_ex_) and 535 nm (λ_em_) for green fluorescence measurements and 540 nm (λ_ex_) and 650 nm (λ_em_) for red fluorescence measurements. Note that the graph in A has a break in the *y*-axis to show the data plotted for strain 2. **(B)** To rule out any effect of the reporter itself on the results, we measured the outputs as in A, but with the following configuration: strain 1, *E. coli* MG1655 carrying J23114-*qdoR*-P_*qdoI*_-*rfp*, and strain 2, *E. coli* MG1655 carrying J23110-*qdoR*-P_*qdoI*_-*gfp*. Q, quercetin. The experiments were conducted with biological triplicates. After induction, the fluorescence of each replicate was measured once at specified times. The error bars represent the standard deviation.

Controlling gene expression at different levels and in different cells simultaneously, adding only one inducer to the culture, is a tool with great biotechnological potential. For example, different gene expression levels resulting from the same inducer concentration are valuable for applications with mixed cultures in reactors (Jawed et al., 2019). However, in a mixture of bacteria expressing high and low levels of an inducer-binding repressor, it is necessary to rule out if cells expressing high levels of the repressor would cause an inducer titration effect. Therefore, to assess whether two populations would respond differently to the same input, we mixed two strains of *E. coli* transformed with different circuits expressing QdoR (Figure 4). Strain 1 carried the low resistance circuit (J23114-*qdoR*), controlling the expression of GFP, whereas strain 2 carried the high resistance circuit (J23110-*qdoR*), controlling the expression of RFP (Scheme in Figure 4A). The two cultures were mixed in the same proportion and induced with different concentrations of quercetin. Both circuits were induced, but as seen before, circuits with higher resistance gave lower output, regardless of the inducer concentration. To verify if the results would be affected by the intrinsic characteristics of each reporter, we exchanged them. The circuits kept the same expression pattern, ruling out any such bias due to the reporters GFP or RFP (Figure 4B).

## Discussion

In this work, we dissected and reassembled the QdoR system of *B. subtilis* in *E. coli* to engineer genetic circuits sensitive to quercetin. Such circuits have different output values to the same input controlling expression of QdoR. We correlated the strength of promoters used and the response curves of GFP expression. We highlight some differences of our circuit to this one reported by Siedler and co-workers (Siedler et al., 2014). We inserted the synthetic RBS B0034 upstream of *qdoR* and *gfp*. The B0034 RBS is a 12 bp sequence of medium strength in protein synthesis compared to the strong RBS used in the Elowitz and Leibler repressilator (Elowitz and Leibler, 2000). Siedler et al. have cloned the native promoters of the *qdoR* and *qdoI*, including the native RBS, which are possibly not recognized, and B0034 by *E. coli* ribosomes. In addition, we noted that in the circuit by Siedler et al., according to the sequence of p441-QdoR, there is no transcriptional terminator additionally inserted downstream of *qdoR* and upstream of the *qdoI* promoter. Eventually, the double terminator that we inserted downstream *qdoR* isolated it from the reporter module (P_*qdoI*_-*gfp*), preventing any transcriptional interference of P_*qdoR*_ over P_*qdoI*_. In addition, it should be noted that p441-QdoR used by Siedler et al. is a pSEVA441 derivative plasmid with the pRO1600/ColE1 origin of replication (Silva-Rocha et al., 2013). ColE1 origin was reported generating 50–70 copies/cell of *E. coli* (Lutz and Bujard, 1997), while the pUC origin as in our plasmids generated ∼500-700 copies/cell. Therefore, the direct comparison of our data with those of Siedler et al. should consider a possible effect of gene dosage on GFP expression. However, as we used the minimum and maximum level of specific fluorescence of both circuits to compare performance, any effect of gene dosage is likely to be already implicit.

A similar dissection and reassembling approach was previously reported in which the FdeR native architecture of *Herbaspirillum seropedicae* was used to construct biosensors responsive to naringenin, with transcriptional factor expression control through different *in silico* designed RBS (De Paepe et al., 2018). However, a linear correlation of the translation initiation rates of the RBS that controlled FdeR synthesis with either the maximum output or the operational range was not found. Compared with our results, varying the strength of the promoter controlling the transcription of *fdeR* could also be an interesting alternative to tune both maximum output and operational range.

By combining the native elements of the native QdoR system with synthetic elements, we designed circuits with varied output values and different noise behavior in the presence or absence of an inducer. We determined that noise is minimal when the repressor QdoR is either mostly free or mostly bound to the inducer. On the other hand, when the level of free QdoR is similar to the level of QdoR bound to quercetin, noise is likely to increase due to transcriptional bursts when a gene is transcribed in a pulse. Negative feedback loops (Savageau, 1974; Becskel and Serrano, 2000) can minimize noise. For example, a circuit with TetR repressing itself and a reporter gene decreased noise compared to a circuit expressing TetR constitutively (Dublanche et al., 2006). Nevozhay et al. (2009) have reported the same, showing that the negative feedback TetR-regulated circuit was less noisy than the circuit expressing TetR constitutively in *S. cerevisiae*. Here, we also showed that noise can be decreased if the repressor is expressed constitutively at high levels, as in the J23110-*qdoR* circuit.

The circuits controlled by QdoR were constructed in high copy number plasmids and showed good reproducibility and stability. Further optimization of the circuits, including reduction of leakiness and increases in the sensitivity and the dynamic range, might be necessary depending on their application. It would also be possible to engineer circuits that have response delays (Hooshangi et al., 2005) or that act as inverters, with high input producing low output. Our research group has a long-term goal of developing gene circuits for plant-bacteria interaction. Such circuits will benefit studies on chemical communication between beneficial rhizobacteria and plants (Pini et al., 2017; Poole, 2017) and aid in designing potential biotech applications. In this sense, the benchmarking of minimal parts, such as the P_*qdoR*_ and P_*qdoI*_ promoters in circuits controlled by quercetin, will be fundamental for bioengineering efficient circuits exploring plant-bacteria communication. For instance, circuits controlled by flavonoids can be applied to control gene expression in bacteria associated with plants, as shown in bacteria expressing genes of the *nif* (nitrogen fixation) cluster when associated with cereals (Ryu et al., 2020).

## Data Availability

The original contributions presented in the study are included in the article/Supplementary Material; further inquiries can be directed to the corresponding author.
