# Supplementary material for: Control of Gene Expression With Quercetin-Responsive Modular Circuits
Source: Front Bioeng Biotechnol. 2021 Sep 16;9:730967. doi: 10.3389/fbioe.2021.730967 (PMC8481877; doi:10.3389/fbioe.2021.730967)
Supplement: Supplementary file 1 [file DataSheet1.docx]

Supplementary Material

Control of gene expression with quercetin-responsive modular circuits

Fernanda Miyuki Kashiwagi^1^, Brenno Wendler Miranda^2^, Fabio de Oliveira Pedrosa^3^, Emanuel Maltempi de Souza^3^, Marcelo Müller-Santos^3*^

*** Correspondence:**

Marcelo Müller-Santos

marcelomuller@ufpr.br

# Supplementary Figures and Tables

## Supplementary Figures


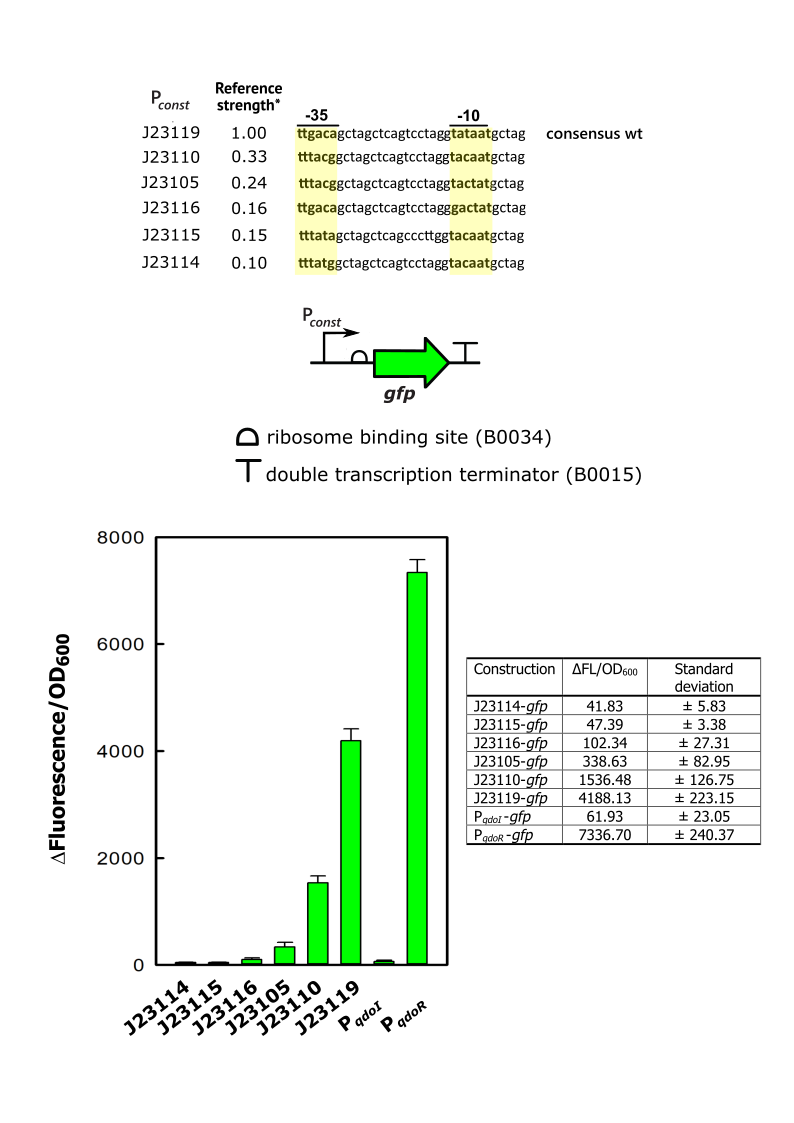


**Supplementary Figure 1.** **Transcription strengths of constitutive promoters in *E. coli* MG1655.** *E. coli* MG1655 was transformed with plasmids bearing a constitutive promoter of the J23XXX series that controlled the expression of *gfp*. * The reference promoter strengths were reported previously in the iGEM dataset (J. Christopher Anderson, http://parts.igem.org/Promoters/Catalog/Anderson) and indicate the strength expected to each promoter selected for the construction of the circuits. The bacteria were cultivated in a 96-well plate as described in the Materials and Methods section. The fluorescence was measured well-to-well every 10 min with λ_ex_ = 485 nm and λ_em_ = 535 nm. The ΔFluorescence/OD_600_ was calculated by linear regression of the Fluorescence × OD_600_ plots in exponential growth. Six constitutive promoters (P*_const_*) with different transcriptional strengths were inserted upstream of *gfp*. The values in the plot are in the table on the side. The promoter sequences are shown, and the -35 and -10 recognition sites are in bold and highlighted in yellow. The J23119 promoter is referred to as the consensus wt. The experiment was conducted with biological triplicates. After induction, the fluorescence of each replicate was measured once at specified times. The error bars represent standard deviation.

Of note, Hirooka et al. (2007) determined that the transcription start site of *qdoI* (*ygaH* in the original publication) was more intense than that of *qdoR* in quercetin-induced *B. subtilis* 168. On the other hand, our results showed that P*_qdoR_* was about 118 times more active than P*_qdoI_* when controlling the expression of GFP. Such difference is probably in part due to the absence of QdoR repression in the experiment shown in Supplementary Figure 1. Furthermore, the -10 and -35 sites of P*_qdoR_* were likely more effectively recognised by σ^70^-RNA polymerase of *E. coli*, resulting in higher GFP levels than expressed by P*_qdoI_*.


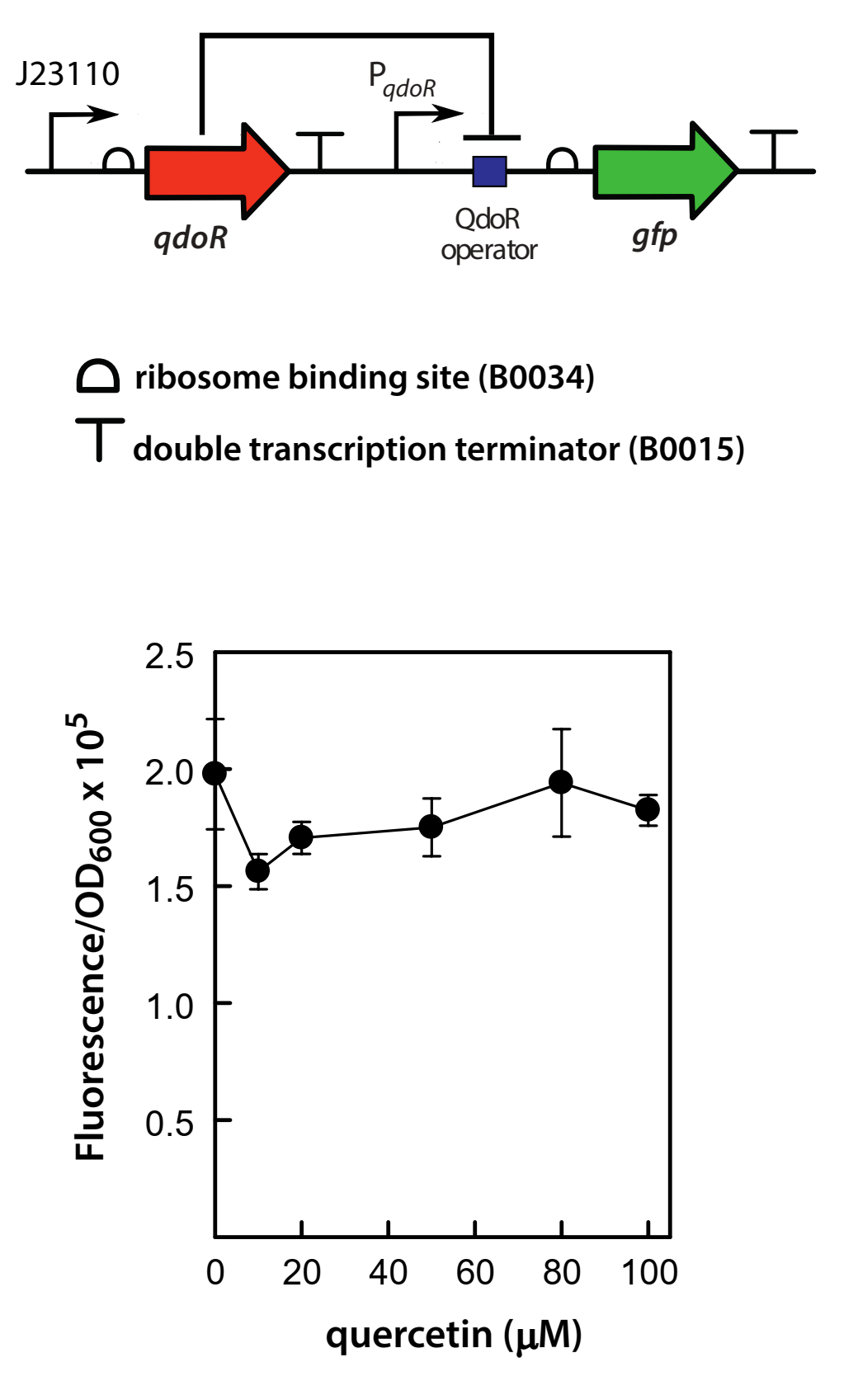


**Supplementary Figure 2**. **GFP expression in the circuit J23110-*qdoR*-P*_qdoR_*-*gfp*.** *E. coli* MG1655 was transformed with the plasmid carrying the circuit J23110-*qdoR*-P*_qdoR_*-*gfp* and grown in a 96-well plate. The culture was induced with increasing concentrations of quercetin. The fluorescence and OD_600_ were measured during 12 h, and the Δfluorescence/OD_600_ was obtained by linear fit of the fluorescence × OD_600_ plots. The experiment was conducted with biological triplicates. After induction, the fluorescence of each replicate was measured once at specified times. The error bars represent standard deviation.


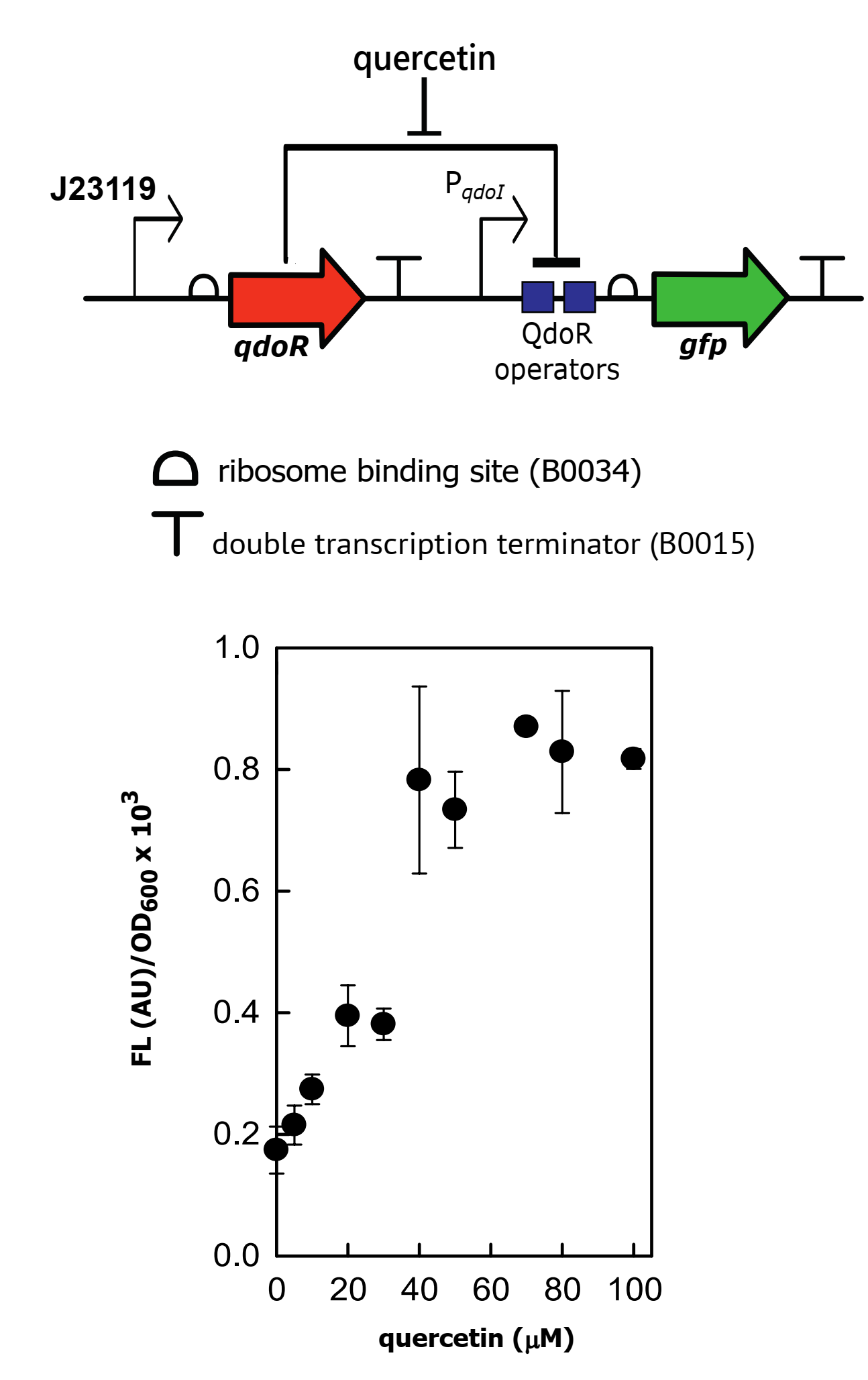


**Supplementary Figure 3**. **GFP expression in the circuit J23119-*qdoR*-P*_qdoI_*-*gfp*.** *E. coli* MG1655 was transformed with the plasmid carrying the circuit J23119-*qdoR*-P*_qdoI_*-*gfp* and grown in a 96-well plate. The culture was induced with increasing concentrations of quercetin. The fluorescence and OD_600_ were measured during 12 h, and the Δfluorescence/OD_600_ was obtained by linear fit of the fluorescence × OD_600_ plots. The experiment was conducted with biological triplicates. After induction, the fluorescence of each replicate was measured once at specified times. The error bars represent standard deviation.


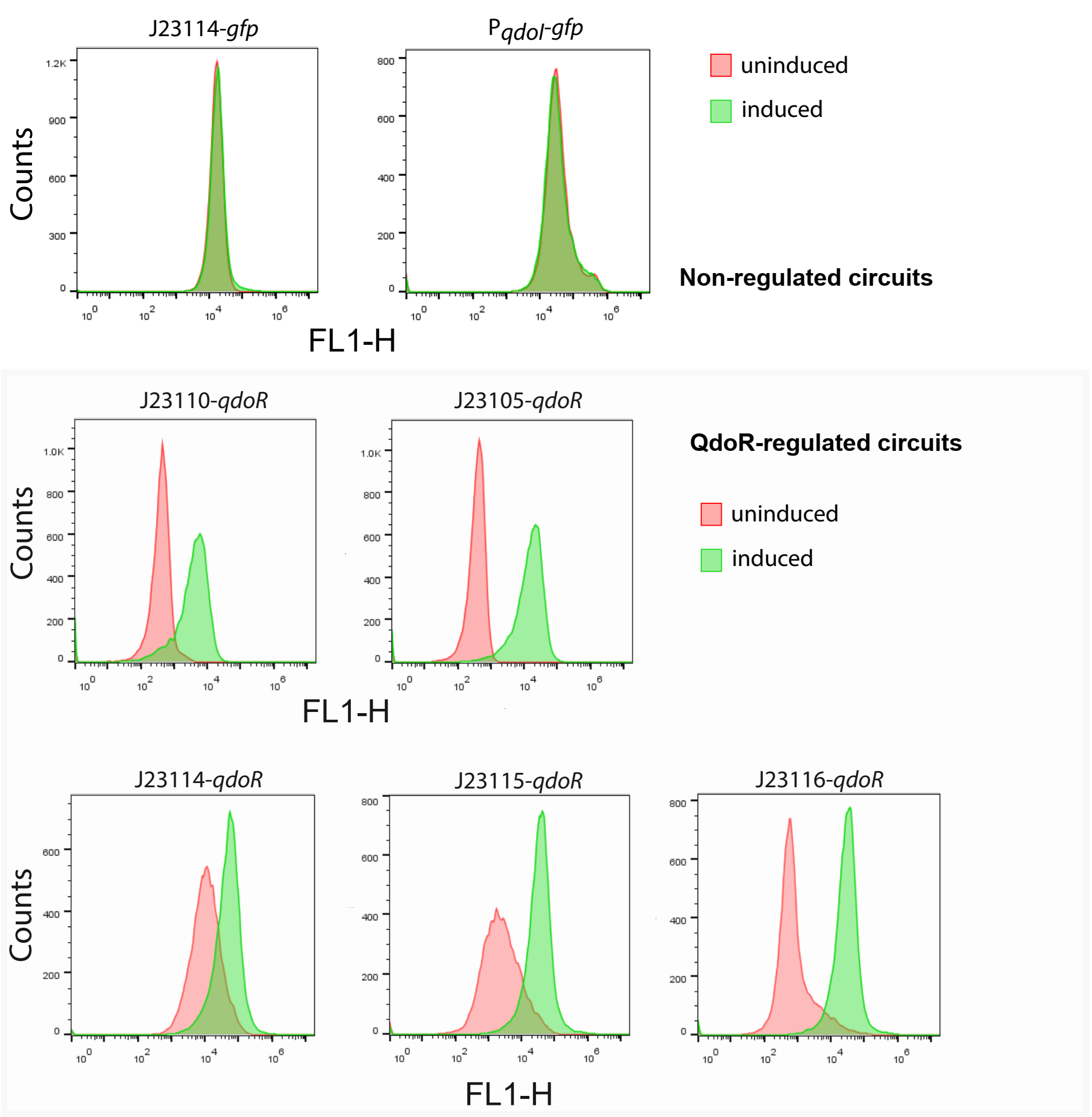


**Supplementary Figure 4**. **Counting of fluorescent *E. coli* cells carrying non-regulated or QdoR-regulated circuits in uninduced and induced states.** *E. coli* MG1655 carrying the circuits described at each graph were grown as described in Materials and Methods. The bacteria were collected and the fluorescence per cell measured before induction (red peaks) and after induction with 100 µM quercetin (green peaks). The fluorescence of GFP expressing bacteria was measured with a filter set at 488 nm excitation and 533 nm of emission. The intensity of fluorescence per cell (FL1-H) by countings was expressed in histograms to each circuit. The experiment was conducted with biological triplicates. After induction, the fluorescence of each replicate was measured once at specified times.


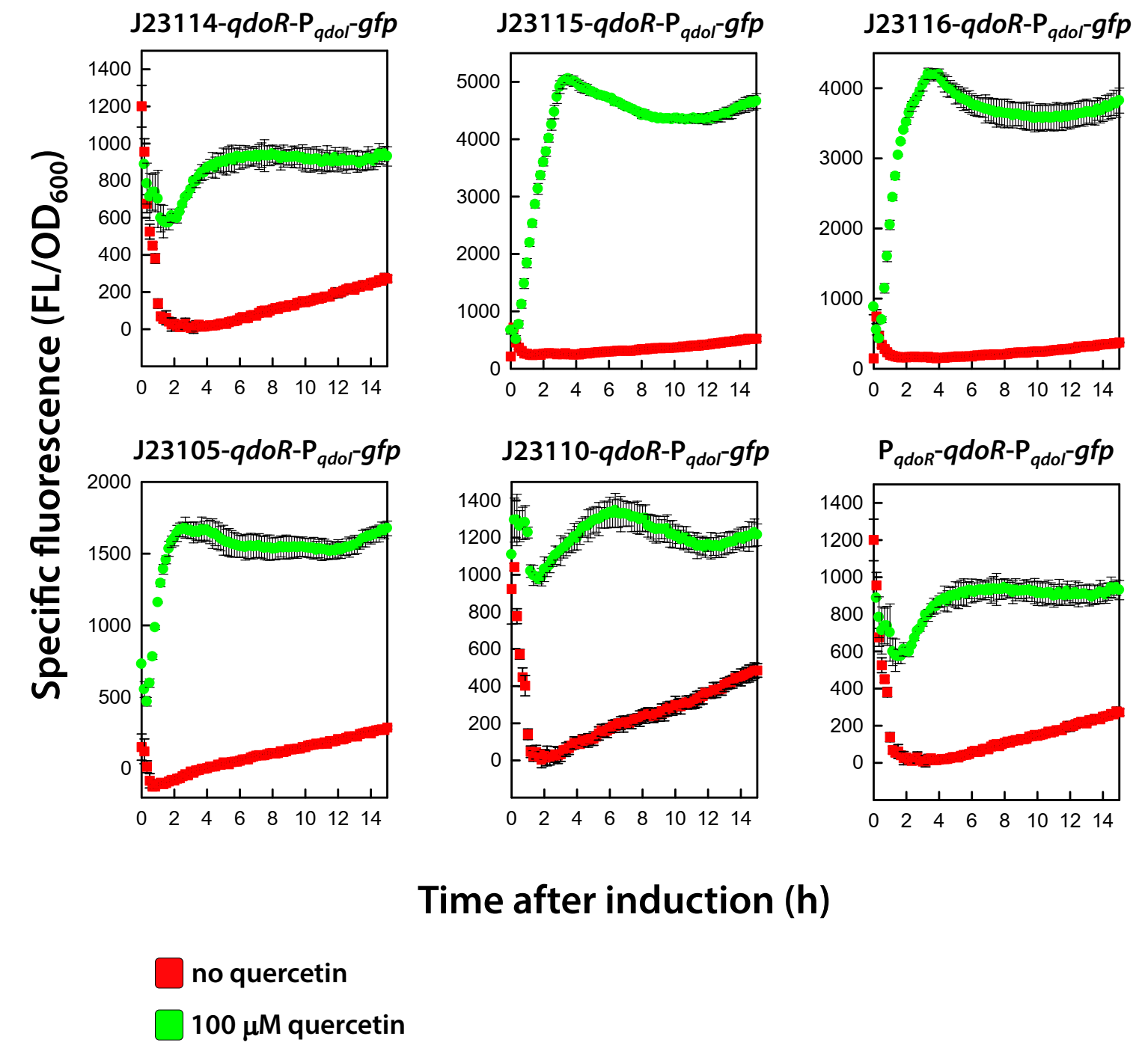


**Supplementary Figure 5. Curves of specific fluorescence of *E. coli* carrying quercetin-responsive circuits after induction.** *E. coli* MG1655 cultures were grown as described in the Material and Methods section. After the addition of quercetin, cultures were grown for 15 h and fluorescence and OD_600_ measured periodically. Here, only the results for induction with 100 µM quercetin are presented. Note that at 6 h after induction, the specific fluorescence values are stable for all induced circuits. Each plot was identified at the top with the name of the analyzed circuit. The experiment was conducted with biological triplicates. After induction, the fluorescence of each replicate was measured once at specified times. The error bars represent standard deviation.

**Supplementary Table 1**. **Sequences of genetic parts used in this study.**

| **P*_qdoI_*^*^** |
| --- |
| *GAATTCGCGGCCGCTTCTAGA*GATTATA**TAGAAC**GGTCTACTCAAATGAAT**GATAAT**AT  **-35** **-10**  ATAGACTGGTCTAT*ACTAGTAGCGGCCGCTGCAG* |
| **P*_qdoR_*^*^** |
| *GAATTCGCGGCCGCTTCTAGA*G**TTAACA**ATCACAGGGTTCTTTTT**TATATT**ATATAAGT  **-35** **-10**  CGCTCTAT*ACTAGTAGCGGCCGCTGCAG* |
| ***qdoR* ^**^** |
| *GAATTCGCGGCCGCTTCTAGA*TGACCTCTCGTGGTGACTCTCGTGAAAAAATCCTGCACACCGCTTCTCGTCTGTTCCAGCTGCAAGGTTACCACGCTACCGGTCTGAACCAGATCGTTAAAGAATCTGGTGCTCCGAAAGGTTCTCTGTACCACTTCTTCCCGAACGGTAAAGAAGAACTGGCTATCGAAGCTGTTACCTACACCGGTAAAATCGTTGAACACCTGATCCAGCAGTCTATGGACGAATCTTCTGACCCGGTTGAAGCTATCCAGCTGTTCATCAAAAAAACCGCTTCTCAGTTCGACAACACCGAATCTATCAAAGGTATCCCGGTTGGTCTGCTGGCTTCTGAAACCGCTCTGATCTCTGAACCGCTGCGTACCGTTTGCATGAAAGTTTTCAAATCTTGGGAAGCTGTTTTCGCTCGTAAACTGATGGAAAACGGTTTCGCTGAAGAAGAAGCTAACCAGCTGGGTACCCTGATCAACTCTATGATCGAAGGTGGTATCATGCTGTCTCTGACCAACAAAGACAAAACCCCGCTGCTGCTGATCGCTGAACAGATCCCGGTTCTGGTTCGTAAAAAAGGTTAAT*ACTAGTAGCGGCCGCTGCAG* |
| **BBa_I13504** |
| AAAGAGGAGAAATACTAGATGCGTAAAGGAGAAGAACTTTTCACTGGAGTTGTCCCAATTCTTGTTGAATTAGATGGTGATGTTAATGGGCACAAATTTTCTGTCAGTGGAGAGGGTGAAGGTGATGCAACATACGGAAAACTTACCCTTAAATTTATTTGCACTACTGGAAAACTACCTGTTCCATGGCCAACACTTGTCACTACTTTCGGTTATGGTGTTCAATGCTTTGCGAGATACCCAGATCATATGAAACAGCATGACTTTTTCAAGAGTGCCATGCCCGAAGGTTATGTACAGGAAAGAACTATATTTTTCAAAGATGACGGGAACTACAAGACACGTGCTGAAGTCAAGTTTGAAGGTGATACCCTTGTTAATAGAATCGAGTTAAAAGGTATTGATTTTAAAGAAGATGGAAACATTCTTGGACACAAATTGGAATACAACTATAACTCACACAATGTATACATCATGGCAGACAAACAAAAGAATGGAATCAAAGTTAACTTCAAAATTAGACACAACATTGAAGATGGAAGCGTTCAACTAGCAGACCATTATCAACAAAATACTCCAATTGGCGATGGCCCTGTCCTTTTACCAGACAACCATTACCTGTCCACACAATCTGCCCTTTCGAAAGATCCCAACGAAAAGAGAGACCACATGGTCCTTCTTGAGTTTGTAACAGCTGCTGGGATTACACATGGCATGGATGAACTATACAAATAATAATACTAGAGCCAGGCATCAAATAAAACGAAAGGCTCAGTCGAAAGACTGGGCCTTTCGTTTTATCTGTTGTTTGTCGGTGAACGCTCTCTACTAGAGTCACACTGGCTCACCTTCGGGTGGGCCTTTCTGCGTTTATA |
| **BBa_K1357010** |
| AAAGAGGAGAAATACTAGATGGCTTCCTCCGAAGACGTTATCAAAGAGTTCATGCGTTTCAAAGTTCGTATGGAAGGTTCCGTTAACGGTCACGAGTTCGAAATCGAAGGTGAAGGTGAAGGTCGTCCGTACGAAGGTACCCAGACCGCTAAACTGAAAGTTACCAAAGGTGGTCCGCTGCCGTTCGCTTGGGACATCCTGTCCCCGCAGTTCCAGTACGGTTCCAAAGCTTACGTTAAACACCCGGCTGACATCCCGGACTACCTGAAACTGTCCTTCCCGGAAGGTTTCAAATGGGAACGTGTTATGAACTTCGAAGACGGTGGTGTTGTTACCGTTACCCAGGACTCCTCCCTGCAAGACGGTGAGTTCATCTACAAAGTTAAACTGCGTGGTACCAACTTCCCGTCCGACGGTCCGGTTATGCAGAAAAAAACCATGGGTTGGGAAGCTTCCACCGAACGTATGTACCCGGAAGACGGTGCTCTGAAAGGTGAAATCAAAATGCGTCTGAAACTGAAAGACGGTGGTCACTACGACGCTGAAGTTAAAACCACCTACATGGCTAAAAAACCGGTTCAGCTGCCGGGTGCTTACAAAACCGACATCAAACTGGACATCACCTCCCACAACGAAGACTACACCATCGTTGAACAGTACGAACGTGCTGAAGGTCGTCACTCCACCGGTGCTTAATAACGCTGATAGTGCTAGTGTAGATCGCTACTAGAGCCAGGCATCAAATAAAACGAAAGGCTCAGTCGAAAGACTGGGCCTTTCGTTTTATCTGTTGTTTGTCGGTGAACGCTCTCTACTAGAGTCACACTGGCTCACCTTCGGGTGGGCCTTTCTGCGTTTATA |
| **BBa_B0034** |
| AAAGAGGAGAAA |
| **BBa_B0015** |
| CCAGGCATCAAATAAAACGAAAGGCTCAGTCGAAAGACTGGGCCTTTCGTTTTATCTGTTGTTTGTCGGTGAACGCTCTCTACTAGAGTCACACTGGCTCACCTTCGGGTGGGCCTTTCTGCGTTTATA |

* QdoR operators highlighted in yellow; -35 and -10 sites according to Hirooka et al. (2007) are in bold; Biobricks prefix and suffix are in italic; in red the bases changed to turn P*_qdoI_* compatible with Biobricks assembly. ** coding sequence is underlined.

**Supplementary Table 2**

Statistical analyses of CV values for circuits at uninduced and induced states. Data presented in Fig. 3B.

| **Circuit 1 (ON/OFF)** | **Circuit 2 (ON/OFF)** | **p-value** |
| --- | --- | --- |
| *Statistical significance between OFF and ON states for the same circuit* | | |
| J23110-*qdoR* (OFF) | J23110-*qdoR* (ON) | *** (0.0001587) |
| J23105-*qdoR* (OFF) | J23105-*qdoR* (ON) | ** (0.01352) |
| J23116-*qdoR* (OFF) | J23116-*qdoR* (ON) | ** (0.001285) |
| J23115-*qdoR* (OFF) | J23115-*qdoR* (ON) | *** (0.0002836) |
| J23114-*qdoR* (OFF) | J23114-*qdoR* (ON) | * (0.03149) |
| J23114-*gfp* (OFF) | J23114-*gfp* (ON) | ns^#^ (0.5626) |
| P*_qdoI_*-*gfp* (OFF) | P*_qdoI_*-*gfp* (ON) | ns (0.08039 |
| *Statistical significance between OFF state for all circuits* | | |
| J23110-*qdoR* (OFF) | J23105-*qdoR* (OFF) | *** (0.0002381) |
| J23110-*qdoR* (OFF) | J23116-*qdoR* (OFF) | ** (0.003012) |
| J23110-*qdoR* (OFF) | J23115-*qdoR* (OFF) | *** (0.0002558) |
| J23110-*qdoR* (OFF) | J23114-*qdoR* (OFF) | * (0.0154) |
| J23110-*qdoR* (OFF) | J23114-*gfp* (OFF) | *** (0.000153) |
| J23110-*qdoR* (OFF) | P*_qdoI_*-*gfp* (OFF) | ** (0.003038) |
| *Statistical significance between ON state for all circuits* | | |
| J23110-*qdoR* (ON) | J23105-*qdoR* (ON) | ns (0.1224) |
| J23110-*qdoR* (ON) | J23116-*qdoR* (ON) | ** (0.004987) |
| J23110-*qdoR* (ON) | J23115-*qdoR* (ON) | * (0.03377) |
| J23110-*qdoR* (ON) | J23114-*qdoR* (ON) | *** (0.000236) |
| J23110-*qdoR* (ON) | J23114-*gfp* (ON) | *** (2.129e-06) |
| J23110-*qdoR* (ON) | P*_qdoI_*-*gfp* (ON) | ns (0.09319) |
| *Statistical significance between medium-* *and low-resistance circuits* | | |
| J23116-*qdoR* (OFF) | J23115-*qdoR* (OFF) | ns (0.1352) |
| J23116-*qdoR* (ON) | J23115-*qdoR* (ON) | ns (0.8379) |
| J23114-*qdoR* (OFF) | J23115-*qdoR* (OFF) | ** (0.004366) |
| J23114-*qdoR* (OFF) | J23116-*qdoR* (OFF) | ** (0.002934) |
| J23114-*qdoR* (ON) | J23115-*qdoR* (ON) | ns (0.2631) |
| J23114-*qdoR* (ON) | J23116-*qdoR* (ON) | ns (0.05688) |

# - ns = no significant difference.

**Supplementary Table 3**

Fluorescence median values for circuits at uninduced and induced states.

Data presented in Fig. 3A.

|  | **Circuits** | | | | | |
| --- | --- | --- | --- | --- | --- | --- |
|  | P*_qdoR_*-*qdoR*-P*_qdoI_*-*gfp* | | J23110-*qdoR*-P*_qdoI_*-*gfp* | | J23114-*qdoR*-P*_qdoI_*-*gfp* | |
| Quercetin (µM) | Median | S.D. | Median | S.D. | Median | S.D. |
| 0 | 373 | 22.07 | 349.33 | 18.47 | 4344.67 | 727.98 |
| 20 | 938 | 150.36 | 512 | 8 | 11028.33 | 547.72 |
| 30 | 1595.67 | 198.63 | 704.33 | 57.35 | 16869.67 | 822.81 |
| 50 | 2838 | 851.36 | 1211.50 | 159.09 | 26104 | 449.72 |
| 80 | 3068.67 | 433.05 | 2036.33 | 173 | 28637.33 | 1234.86 |
| 100 | 3529.67 | 404.78 | 2336.33 | 313.15 | 30832.33 | 332.04 |

S.D. – standard deviation.

Data presented in Fig. 3B.

|  | 0 µM quercetin | | 100 µM quercetin | |
| --- | --- | --- | --- | --- |
|  | Median | S.D. | Median | S.D. |
| J23110-*qdoR*-P*_qdoI_*-*gfp* | 349.33 | 18.47 | 2336.33 | 313.15 |
| J23105-*qdoR*-P*_qdoI_*-*gfp* | 400 | 20.81 | 13789.33 | 1420.72 |
| J23116-*qdoR*-P*_qdoI_*-*gfp* | 595 | 16.97 | 29280.5 | 2015.96 |
| J23115-*qdoR*-P*_qdoI_*-*gfp* | 2125.33 | 82.35 | 32838 | 660.44 |
| J23114-*qdoR*-P*_qdoI_*-*gfp* | 4344.67 | 727.98 | 30832.33 | 332.04 |
| J23114-*gfp* | 16178.67 | 1363.09 | 15971.33 | 403.21 |
| P*_qdoI_*-*gfp* | 30017.33 | 926.47 | 26759.333 | 1037.38 |
